# The Neurobiology of Selenium: Looking Back and to the Future

**DOI:** 10.3389/fnins.2021.652099

**Published:** 2021-02-25

**Authors:** Ulrich Schweizer, Simon Bohleber, Wenchao Zhao, Noelia Fradejas-Villar

**Affiliations:** Institut für Biochemie und Molekularbiologie, Medizinische Fakultät, Rheinische Friedrich-Wilhelms-Universität Bonn, Bonn, Germany

**Keywords:** genetics, neurodegeneration, GPX4, ferroptosis, epilepsy

## Abstract

Eighteen years ago, unexpected epileptic seizures in *Selenop*-knockout mice pointed to a potentially novel, possibly underestimated, and previously difficult to study role of selenium (Se) in the mammalian brain. This mouse model was the key to open the field of molecular mechanisms, i.e., to delineate the roles of selenium and individual selenoproteins in the brain, and answer specific questions like: how does Se enter the brain; which processes and which cell types are dependent on selenoproteins; and, what are the individual roles of selenoproteins in the brain? Many of these questions have been answered and much progress is being made to fill remaining gaps. Mouse and human genetics have together boosted the field tremendously, in addition to traditional biochemistry and cell biology. As always, new questions have become apparent or more pressing with solving older questions. We will briefly summarize what we know about selenoproteins in the human brain, glance over to the mouse as a useful model, and then discuss new questions and directions the field might take in the next 18 years.

## A Brief History of the Field

When selenoprotein P (*Selenop*)-knockout mice were made independently in two laboratories, it was not expected that their most dramatic phenotype was to reveal the essential requirement of selenium (Se) in the brain (Hill et al., 2003; Schomburg et al., 2003). However, the model was tricky: the neurological phenotype depended exquisitely on the level of dietary Se supply and, more precisely, on the timing of Se deficiency during the ontogeny of the animal. The neurological phenotype varied between none at all, epileptic seizures, movement phenotype with ataxia and/or dystonia, overt neurodegeneration with premature death, or death before weaning (Hill et al., 2004; Schweizer et al., 2004b, 2005; Valentine et al., 2005; Schweizer, 2016). Since Se levels in commercial diets may not always precisely reflect the printed specifications in every lot of mouse chow, and because the *Selenop*-knockout mice reflected so sensitively the dietary Se supply, working with the model was difficult, to say the least. At the time, antibodies against selenoproteins were not widely available, and enzymatic assays for glutathione peroxidase (GPX) and thioredoxin reductase (TXNRD), Se measurements, and metabolic labeling with ^75^Se were the methods of choice. At least, these methods clearly demonstrated, that inactivation of *Selenop*, a gene mostly expressed in liver and secreted into the plasma, reduced Se levels and selenoenzyme activities in the brain to a degree impossible to achieve with dietary Se restriction alone (Hill et al., 2003; Schomburg et al., 2003). New selenoproteins were still being discovered, until a landmark genomic study fixed the number at the final 25 in humans and 24 in mice (Kryukov et al., 2003). In an early review (Schweizer et al., 2004c), the key questions about the neurobiology of Se were: Which selenoproteins are expressed in the brain, in which regions and in which cell types? What are their functions? How does Se enter the brain and is it distributed in a hierarchical manner? Is there a causal connection to neurological disorders?

To cut the answers short: most selenoproteins are expressed in the brain, mainly in neurons, in all major brain regions (Zhang et al., 2008). Gene targeting in mice for all or single selenoproteins has revealed that GABAergic interneurons are particularly vulnerable, but also basal ganglia, cerebellum, cortex, and brain stem (Valentine et al., 2005; Wirth et al., 2010, 2014; Pitts et al., 2012; Seeher et al., 2014). At first it remained an open question whether myelination defects were caused by neuronal or oligodendroglial selenoprotein dysfunction (Valentine et al., 2008). Several selenoproteins are essential for the brain development and function, in particular glutathione peroxidase 4 (GPX4), but also thioredoxin reductase 1 (TXNRD1), and SELENOT (Castex et al., 2015). Currently the function of GPX4 as a major regulator of ferroptosis in development and disease is receiving a great deal of attention (Stockwell et al., 2017; Friedmann Angeli et al., 2019). It will be seen whether GPX4 is an indispensable protein for neurons in its own right or whether there is a causal connection to neurodegeneration in neurological disorders. What is now clear, is that Se enters the brain either as non-physiological selenite salt or, more physiologically, in the form of SELENOP, loaded in the liver with several atoms of Se (Schweizer et al., 2005; Renko et al., 2008; Hill et al., 2012), and taken up at the blood-brain-barrier (BBB) and by individual cells via endocytosis using receptors of the LRP family (Burk and Hill, 2015). The most important receptor is APOER2 (Olson et al., 2007), but also LRP2/MEGALIN (Olson et al., 2008; Chiu-Ugalde et al., 2010) and, most likely, LRP1 contribute to SELENOP internalization. Among these receptors, APOER2 clearly is the most important, providing preferential Se supply at the BBB and in the testis. MEGALIN appears more involved in Se supply through the blood-cerebrospinal fluid barrier and in the kidney (Burk and Hill, 2015). *SELENOP* is also expressed in the brain and may contribute to local Se storage and recycling (Scharpf et al., 2007; Renko et al., 2008). SELENOP and its receptors contribute to a “hierarchy” of selenoprotein expression in organs in the sense that some organs, like the brain, can be preferentially supplied with Se at the expense of others, like the liver. There is, however, a second “hierarchy” of selenoprotein expression that depends on the relative sensitivity of individual selenoproteins to Se availability. Thus, GPX1 is more sensitive to cellular Se levels than, e.g., GPX4. The reasons behind this observation are multifarious and beyond the scope of this review. Both hierarchies may work hand-in-hand as *GPX1* mRNA is highly expressed in liver and contains a highly efficient SECIS element. Depending on dietary Se supply, the amount of GPX1 protein covers orders of magnitude, and likely provides a safe Se storage device, until the Se is needed for distribution via SELENOP to preferred target tissues. The amount of Se in a tissue does not necessarily inform about its physiological importance, e.g., brain Se levels are much lower than liver Se levels, yet selenoproteins are essential in brain, but not liver (Schweizer et al., 2005; Wirth et al., 2010).

## Genetic Deficiency of Single Selenoproteins

At that time, only one human disorder was genetically linked to deficiency of a selenoprotein-encoding gene, now known as selenoprotein N (SELENON)-related myopathies (Moghadaszadeh et al., 2001; Castets et al., 2012), but the situation was soon to change with exome sequencing entering clinical practice. SELENON is an ER-resident membrane protein of unknown function. Mutations in *SELENON* that disrupt the gene or prevent selenocysteine (Sec) insertion into the protein lead to myopathy (Villar-Quiles et al., 2020). Mouse and zebrafish models of SELENON-deficiency reflect aspects of the muscular phenotype, in particular the preferential affection of axial muscles (Rederstorff et al., 2011). This example suggests that the mouse may represent an acceptable model for humans regarding selenoprotein deficiency. It should be noted that dietary deficiency for Se in livestock was recognized early as a cause for white muscle disease (Muth et al., 1958). So far there is no evidence to link *SELENON* mutations to impaired neuronal or neurological function.

The syndromes associated with selenoprotein deficiency can be broadly divided into two categories: the first category represents mutations in single genes encoding selenoproteins. The second category is represented by mutations in genes involved in selenoprotein biosynthesis. A comprehensive compilation of individual mutations and patient phenotypes can be found in recent summaries of the state of the field (Schweizer and Fradejas-Villar, 2016; Fradejas-Villar, 2018; Schoenmakers and Chatterjee, 2020). Here, we will only briefly summarize these results and rather focus on new developments since then.

Conditional gene inactivation of *Gpx4* in mice demonstrated that GPX4 is an essential selenoprotein for several types of neurons as discussed above (Seiler et al., 2008). Conditional inactivation of *Gpx4* in forebrain neurons after development lead to cognitive decline and hippocampal neurodegeneration (Hambright et al., 2017). Furthermore, constitutive gene inactivation of *Gpx4* lead to embryonic lethality around embryonic day 7 (Yant et al., 2003; Seiler et al., 2008). Thus, it came as a complete surprise to find newborn children affected with Sedaghatian-type spondylometaphyseal dysplasia with inactivating non-sense mutations in the *GPX4* gene (Aygun et al., 2012; Smith et al., 2014). These patients show massive brain atrophy and usually die shortly after birth. In stark contrast to mice, where *Gpx4*-deficiency leads to early embryonic lethality, human fetuses obviously progress much further in their development.

A lesser sensitivity of humans compared to mice regarding the lack of selenoproteins was also observed with respect to TXNRD2. *Txnrd2*^–/–^ mice died around embryonic day 13 with thinned ventricular walls in the heart and impaired hematopoiesis (Conrad et al., 2004). Conditional ablation of *Txnrd2* in the heart lead to a fatal cardiomyopathy (Conrad et al., 2004). Similarly, heterozygous missense mutations were associated with dilated cardiomyopathy in humans (Sibbing et al., 2011) and were reminiscent of Keshan disease, a fatal cardiomyopathy observed in Se-deficient regions in China (Ge et al., 1983; Loscalzo, 2014). It thus came as a complete surprise that a homozygous truncating mutation in human *TXNRD2* merely resulted in familial glucocorticoid deficiency without a cardiac phenotype (Prasad et al., 2014).

Similar to inactivation of *Txnrd2*, constitutive inactivation of *Txnrd1* is embryonic lethal in mice (Jakupoglu et al., 2005). Conditional ablation of *Txnrd1* in neuronal precursors shows only a mild cerebellar defect (Soerensen et al., 2008), while neuron-specific *Txnrd1* ablation leads to neurodegeneration with aging (Schweizer and Schomburg, 2006). Since our last review of this subject, we have found that homozygous mutations in *TXNRD1*, which reduce enzymatic activity, are associated with genetic generalized epilepsy in human (Kudin et al., 2017). In summary, upon *Txnrd* mutation the mouse phenotypes appear throughout more severe than the corresponding human phenotypes. This raises the question whether variants of TXNRDs may be able to compensate for the loss of the other TXNRD in humans, but not in mice, or whether, e.g., the glutaredoxin system may be able to partially compensate in some human cell types.

Ethanolamine-phosphotransferase 1 (EPT1/SELENOI) is one of two enzymes catalyzing the same step in phospholipid biosynthesis (Gladyshev et al., 2016). This enzyme is obviously important for myelin biosynthesis in the human brain (Ahmed et al., 2017; Horibata et al., 2018). Inactivation of the gene in mice is embryonic lethal (Avery et al., 2020). Se deficiency of the brains of *Selenop*-deficient mice impaired the myelin sheath in the brain stem, at least under conditions of low Se diet (Valentine et al., 2005). Thus, it is possible that the myelination defect is a primary phenotype of myelin formation in oligodendrocytes and not a result of retrograde signaling from selenoprotein-deficient neurons, as we initially suspected.

The only other selenoprotein that has been shown to play an essential role in the brain is SELENOT (Castex et al., 2015; Boukhzar et al., 2016). How this relates to its role in protein glycosylation is not clear, yet (Hamieh et al., 2017). It is intriguing, that other selenoproteins have also been implicated in protein glycosylation or protein folding, e.g., SELENOF and SELENOM. The respective knockout mouse models, however, did not show any apparent neurological defects (Kasaikina et al., 2011). SELENOS, another selenoprotein implicated in endoplasmic reticulum associated degradation of proteins (ERAD) (Curran et al., 2005), has not been studied by gene targeting in mice. While selenoproteins have often simply been classified as “anti-oxidant,” it is remarkable that inactivation of a selenoenzyme with a defined reductase activity, methionine-R-sulfoxide-reductase B1 (MSRB1), has not produced a neurological phenotype (Fomenko et al., 2008; Lee et al., 2013).

There is a rich literature on mouse models of neurodegenerative diseases whose phenotypes can be exacerbated by additional deficiency of “antioxidant” selenoproteins (Schweizer et al., 2004a; Zhang et al., 2020). In such models there is always the conceptual question whether the mutation in the selenoprotein specifically abrogates (and thus reveals) a specific protective mechanism or whether the selenoprotein mutation simply tips over a dysbalanced system that is already vulnerable to any other possible stressor. Given the availability of many powerful genome-wide association studies on important neurodegenerative disorders, and their failure to identify mutations in selenoprotein genes, it seems unlikely for us that mutations in selenoproteins are important causes or modifiers of common neurological disorders. Yet, mutations in selenoproteins or their biosynthesis pathways may reveal specific cell biological or developmental functions of selenoproteins.

## Selenoprotein Deficiency Resulting From Mutations Impairing Selenoprotein Biosynthesis

**FIGURE 1:**
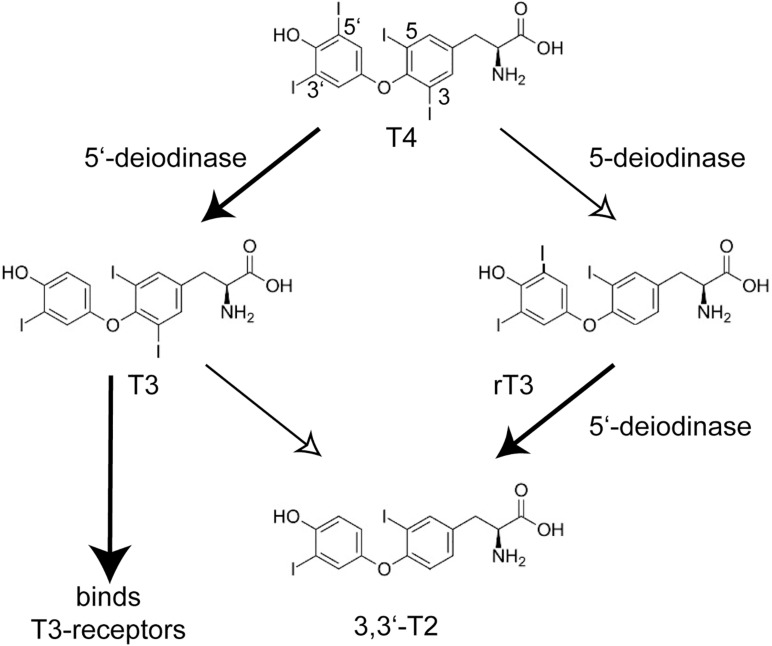
Conversion of iodothyronines by deiodinases and activation of the nuclear T3-receptors. The main product of the thyroid gland is thyroxine (T4, 3,3′,5,5′-tetraiodothyronine). The actions of 5′-deiodinases (DIO1 and DIO2) and 5-deiodinases (DIO1 and DIO3) lead to T3 (3,3′,5-triiodothyronine) and rT3 (3,3′,5′-triiodothyronine), respectively. Only T3 activates the nuclear T3-receptors TRα and TRβ. T2 and rT3 are thus inactive metabolites and a cell can shape its local T3 level through DIO expression. Inactivation of *Dio1* as well as *SECISBP2*-deficiency lead to increased plasma rT3.

A landmark paper on the identification of mutations in the selenoprotein biosynthesis factor SECISBP2 in humans called into question the possibly simple-minded concept of selenoproteins as “anti-oxidants.” The key phenotype that brought the patients to medical attention, was a growth retardation in puberty (Dumitrescu et al., 2005). Abnormal thyroid function tests (TFT), i.e., the constellation of thyroid-stimulating hormone (TSH) and thyroid hormone levels, guided the discovery of a congenital deficiency of selenoprotein biosynthesis. The pubertal growth spurt depends not only on growth hormone, but requires permissive action of thyroid hormone. The TFT suggested deficiency of deiodinase 2 (DIO2) activity in these patients which was confirmed in patient fibroblasts. Deiodinases are selenoenzymes capable of removing iodide from iodothyronines (Köhrle et al., 2005; Mondal et al., 2016). The prohormone thyroxine (T4) requires 5′-deiodination to yield triiodothyronine (T3), which binds the nuclear T3-receptors (Figure 1). 5-deiodination of T4 and T3 yields reverse T3 (rT3) and 3,3′-T2, respectively. Moreover, the two plasma selenoproteins SELENOP and GPX3 were reduced in these patients (Dumitrescu et al., 2005). Thus, the congenital deficiency of selenoprotein biosynthesis revealed itself not in neurodegeneration, epilepsy, heart disease or a muscular disorder, but in altered thyroid hormone levels in the sense of a blunted response to T4! Later, more patients with apparently stronger mutations in SECISBP2 were identified (Di Cosmo et al., 2009; Azevedo et al., 2010; Schoenmakers et al., 2010). Some of these patients exhibited a SELENON-related myopathy, infertility, and an immune phenotype (Schoenmakers and Chatterjee, 2018). The importance of local conversion of T3 is illustrated by the Thr92Ala polymorphism in DIO2. People with the homozygous Ala92 version of this polymorphism have a reduced ability to convert T4 to T3 (McAninch et al., 2015), hence when being treated for hypothyroidism, have improved psychological well-being on combination T4/T3 therapy than on T4 treatment alone (Bianco and Kim, 2018). Remarkably, the first patient with a mutation in the tRNA^*Sec*^ gene (*TCA-TRU* in human, *Trsp* in mouse) showed the same phenotype of a blunted response to T4 (Schoenmakers et al., 2016). Findings from *Dio1-* and *Dio2-*knock-out mouse models are entirely compatible with the above conclusions drawn from *SECISBP2*-deficiency (Schneider et al., 2001, 2006). Selenoprotein deficiency does not fundamentally impair thyroid gland function (Chiu-Ugalde et al., 2012).

This leads us to the obvious question whether other factors involved in selenoprotein biosynthesis have been found mutated in humans. And what kind of phenotypes are presented by affected individuals? The first patients with mutations in the selenocysteine synthase gene (*SEPSECS*) have been identified in Agamy et al. (2010). The patients presented with “progressive cerebello cerebral atrophy,” now systematically designated pontocerebellar hypoplasia 2D (PCH2D). The names of the syndromes capture quite well the observed phenotypes (Schoenmakers and Chatterjee, 2020). The predominantly neurological condition with neurodegeneration and epilepsy is likely based on dysfunction of GPX4 and other essential selenoproteins, possibly TXNRD1 or 2 (Anttonen et al., 2015). Some patients display milder phenotypes and may grow into adulthood with intellectual disability, but no overt neurodegeneration (Iwama et al., 2016). Interestingly, we are not aware of reports of abnormal TFT in these patients. Likewise, we do not know of a *SELENON*-related myopathy in one of these patients.

**FIGURE 2:**
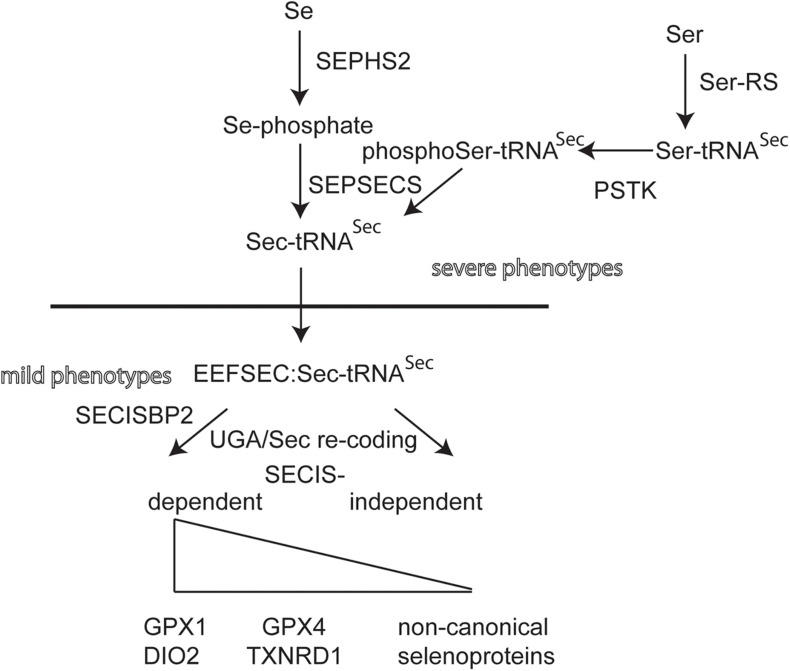
Biosynthetic pathway of selenoprotein translation. Transfer-RNA^*Sec*^ is charged with Ser by Seryl-tRNA synthase (Ser-RS), hence the more accurate designation tRNA^*[Ser]Sec*^. The kinase PSTK phosphorylates Ser-tRNA^*Sec*^. Selenophosphate synthase (SEPHS2) provides selenophosphate which is used by selenocysteine synthase (SEPSECS) to convert phosphoSer-tRNA^*Sec*^ into Sec-tRNA^*Sec*^. EEFSEC is a translation elongation factor specific for Sec-tRNA^*Sec*^. Canonical selenoproteins carry a selenocysteine insertion sequence (SECIS) in their mRNA in order to re-code the UGA codon as Sec codon. The dependence of UGA/Sec re-coding varies among canonical selenoproteins. Several non-canonical selenoprotein genes have been described that do not contain a SECIS element (Guo et al., 2018).

Individuals with mutations in *EEFSEC*, *SEPHS2*, and *PSTK* have not yet been found (Figure 2). Selenoprotein expression in knockout mouse models for these genes have not been described. Conditional inactivation of the suggested biosynthesis factor SECP43, encoded by the *Trnau1ap* gene, in liver and in neurons did not support a role for this gene in selenoprotein expression (Mahdi et al., 2015). Mouse models for hypomorphic mutations in the tRNA^*Sec*^ have been generated. One model has a mutation in the promoter and, as a simple transgene, is inserted somewhere in the genome (Carlson et al., 2009). This mouse displays a neurological phenotype that resembles in several aspects of *Selenop-* and neuron-specific *Trsp-*knockout mutants (Schomburg et al., 2003; Wirth et al., 2010, 2014). Point mutations have been made in *Trsp* affecting the anticodon loop of tRNA^*Sec*^. Overexpression of the A37G-*Trsp* mutant (made as a simple transgene) resulted in neurological defects, in particular when fed a high Se diet (Kasaikina et al., 2013). TFTs have not been determined for the *Trsp* mutant mouse models.

Looking at these results, there seems to exist a strange dichotomy of phenotypes being related either to neurobiology or endocrinology, when selenoprotein biosynthesis is impaired. This observation holds for both mice and humans. A naïve epistatic model of selenoprotein biosynthesis should predict more or less the same phenotypes, if selenoprotein translation is globally impaired (Figure 2). Clearly, we have not yet analyzed all possible mutants and not all available mutants have been systematically analyzed side by side. Yet, in humans, mutations most often come as missense, splicing or other mutations that may not completely abrogate 100% of gene/protein expression/activity. We have recently shown that the effect of a missense mutation *in vitro* and *in vivo* may differ, in particular stability of a mutated protein may depend on the cell type (Zhao et al., 2019). For the SECISBP2^*R*543*Q*^ mutant, we demonstrated that the protein is a complete *NULL* in mouse liver, but partially functioning and supporting selenoprotein expression in neurons (Zhao et al., 2019).

## Future Directions

In order to understand the neurobiology of Se, we need both, the precise biochemical or cell biological function of each selenoprotein and the full understanding of the phenotypes under conditions of its absence in an entire mammalian organism. This goal has only been achieved for a small subset of selenoproteins. For some of the others, we may have a biochemical reaction and a phenotype of cells grown in a dish, but we are convinced that nobody would have been able to predict the complex phenotype of patients with mutations in *SECISBP2* based on the finding of reduced selenoprotein expression in *SECISBP2*-deficient cells in culture. If we just focus on the brain with its many neuronal and glial cell types, we are confronted with perplexing complexity (Zhang et al., 2008). All of these cell types are involved in mechanisms of development, exert a function in the mature organism, and may play are role in neurodegeneration. Thus, it is obvious how wide this field still is and how much expertise is required to address this question.

Another question related to the discussion above, is to what extent mice are valid models for humans with regard to understanding the functions of selenoproteins. The answer will again rely on the comparison of genetic models in mice and patients with congenital defects in selenoprotein genes or genes encoding selenoprotein biosynthesis factors. We can expect that exome-sequencing approaches that are now broadly available will help us identify patients with such mutations. A recent thought-provoking paper has looked at the same question from just the opposite perspective: Instead of searching for mutations in the genes of patients with clinical phenotypes, Santesmasses et al. (2020) searched human genome data for inactivating mutations in selenoprotein genes. They found that humans can carry homozygous inactivating mutations in *SELENOO* without apparently presenting with a phenotype. SELENOO is a novel mitochondrial protein Ser/Thr-AMP transferase that has not yet been inactivated in mice (Sreelatha et al., 2018).

The question whether the selenoproteome is completely known seemed to have been solved through the landmark paper by Kryukov et al. (2003) who identified genes encoding selenoproteins based in part on the presence of the SECIS element. A recent proteome paper now suggested there are additional Sec-containing proteins with UGA/Sec codons, but lacking recognizable SECIS elements (Guo et al., 2018). This provocative finding is, interestingly, in line with the demonstration of selenoprotein translation in the absence of functional SECISBP2 (Fradejas-Villar et al., 2017; Zhao et al., 2019). If mutations in *SEPSECS*, unlike mutations in *SECISBP2*, would also affect selenoproteins that do not depend on a SECIS for biosynthesis, the dichotomy of phenotypes could be explained and some of the non-canonical selenoproteins would likely be important for the brain.

The arguably most dynamic field of selenoprotein research, again related to the neurobiology of Se, is the wider context of the function of GPX4. The whole field of ferroptosis is blossoming. This type of cell death emerges as an important cell biological process on which much hope is placed in the context of cancer treatment and prevention of neurodegenerative disease. Can ferroptosis be modulated pharmacologically to the benefit of patients? Do other pathways related to selenoproteins play a role in these processes? What is the role of lipid peroxidation in this context? In mitochondria? This reminds one of us (US) of a lab rotation in organic chemistry long ago: during his undergraduate study he separated lipid-hydroperoxides and their respective alcohols on a chiral gas-chromatographic column and observed that the peroxides and alcohols were chiral. An enzymatic process seemed the most likely explanation, while biologists argued that spontaneous lipid peroxidation was most likely overinterpreted… Keeping this in mind, who knows what exciting findings there are just around the corner revealing a part of themselves seemingly as oddities or artifacts?

## Author Contributions

US wrote the initial draft of the manuscript. All authors refined and rewrote parts of the manuscript and contributed to research associated with the thoughts presented here.

## Conflict of Interest

The authors declare that the research was conducted in the absence of any commercial or financial relationships that could be construed as a potential conflict of interest.
